# Perioperative Complications of Transvenous Embolization of Ruptured Intracranial Arteriovenous Malformations

**DOI:** 10.3389/fneur.2022.873186

**Published:** 2022-03-30

**Authors:** Yanyan He, Weixing Bai, Bin Xu, Xiaoyu Kang, Jiangyu Xue, Yingkun He, Tianxiao Li

**Affiliations:** ^1^Department of Cerebrovascular Disease and Neurosurgery, Zhengzhou University People's Hospital, Zhengzhou, China; ^2^Department of Cerebrovascular Disease and Neurosurgery, Henan University People's Hospital, Zhengzhou, China; ^3^Department of Cerebrovascular Disease and Neurosurgery, Henan Provincial People's Hospital, Zhengzhou, China; ^4^Henan Provincial NeuroInterventional Engineering Research Center, Henan International Joint Laboratory of Cerebrovascular Disease, and Henan Engineering Research Center of Cerebrovascular Intervention Innovation, Zhengzhou, China

**Keywords:** intracranial arteriovenous malformations, transvenous embolization, complications, cerebral hemorrhage, cerebral infarction

## Abstract

**Purpose:**

To investigate the perioperative complications of transvenous embolization of ruptured intracranial arteriovenous malformations.

**Materials and Methods:**

A total of 27 patients with ruptured intracranial arteriovenous malformations underwent transvenous embolization were enrolled from November 2016 to May 2020 in our prospective database. Perioperative complications and angiographic characteristics were analyzed retrospectively.

**Results:**

Complete disappearance of the nidus occured in 22 (88%) of 25 patients with technically feasible AVMs immediately after embolization. Two cases were partially treated by transarterial embolization due to the failure of microcatheter placement into the draining vein. Seven (25.9%, 7/27) patients had perioperative complications, including three cases of intraoperative hemorrhage, three cases of postoperative hemorrhage and one case of ischemic infarction. No significant differences in complication rates between patients with nidus ≥3 cm and <3 cm (*P* = 0.659), eloquent area and non-eloquent (*P* = 0.137), deep location and superficial (*P* = 0.637), deep venous drainage and cortical vein (*P* = 1.0), the number of venous drainage (*P* = 0.49), the angle of draining vein entering venous sinus <90° and ≥90° (*P* = 1.0), aneurysms (*P* = 0.058) and the time between hemorrhage and TVE (*P* = 1.0) were found. Three of these patients received ventriculostomy, two of which received lumbar drainage treatments at the same time, and four patients just received conservative management. Good outcomes (mRS ≤ 2) at the 1-month evaluation were achieved in 5 of the patients who had complications, but poor outcome (mRS = 5) at the 1-month evaluation was in 1 patient, and 1 lethal complication occurred.

**Conclusion:**

The most common complication of AVMs with transvenous endovascular embolization is cerebral hemorrhage. The prevention of complications may improve the efficacy of AVM embolization, but the current quality of evidence is low and limited in guiding policy development and improving the TVE for AVMs. It is, therefore, necessary to develop clinical research programs in this field.

## Introduction

Intracranial arteriovenous malformations (AVMs) are common in young adults. The first symptoms are mainly hemorrhage and epilepsy, with an AVM annual hemorrhage rate of 1–33% (1). The cure rate of transarterial endovascular embolization of AVMs is about 23% (2). With the improvement in the embolization techniques, materials, and concept, successful cases of transvenous endovascular embolization (TVE) for AVMs have been reported continuously, with a cure rate of 80–100% in single-center literature reports (3–9). However, at present, only a few cases are treated with TVE, and hence, the safety of the TVE needs to be confirmed. Specifically, our previous studies suggested that the incidence of complications was as high as 24%. Thus, exploring the complications of TVE for the treatment of AVMs is essential. A total of 27 patients with AVMs treated by TVE enrolled in our center were analyzed in this prospective study, and the occurrence, pathogenesis, preventive measures, and treatments of the perioperative complications were investigated to provide reference and experience for AVMs treated by TVE, in order to achieve high cure rate and low complication rate.

## Materials and Methods

The Institutional Ethics Committee approved this study. Key inclusion criteria were as follows: ① Patients with a ruptured brain AVM. ② Patients not suitable for transarterial embolization due to the absence of arterial access, narrow arterial feeders, extremely tortuous course, too many feeders, and so forth; ③ Patients in which lesions were not amenable to surgery or radiosurgery, or patients who refused to undergo surgery or radiosurgery. ④ Patients with favorable venous angioarchitecture (Exclusive main draining veins collector which facilitates to navigate microcatheter from sinus to the draining vein) and single main draining vein.

Key exclusion criteria were as follows: ① Multiple AVMs; ② Patients with two or more main draining veins; ③ History of severe allergies to contrast or other non-adhesive embolic agents; ④ Uncontrolled active bleeding.

All the embolization procedures were performed under general anesthesia. A 6-F sheath was placed in the internal jugular vein followed by a 6-F guiding catheter which was advanced to the main draining vein of the brain AVMs. One or two microcatheters [Marathon (Medtronic, USA); Apollo (Medtronic); Echelon (Medtronic); or Headway DUO (MicroVention, Inc., USA)] were placed as close as possible to the nidus of AVM. A vascular sheath was placed in the right femoral artery followed by guide catheter placement though which a microcatheter was advanced into the feeding artery of the AVM. Arterial inflow of feeding artery was reduced by transarterial coil or liquid embolization, or balloon inflation. Transvenous embolization was initiated by injecting ethylene vinyl copolymer (Onyx, Medtronic, California, USA) into the nidus through the venous access route. Transvenous partial coiling in the draining vein through one microcatheter (known as transvenous pressure cooker technique) was used to prevent reflux of Onyx (10, 11). At the completion of the procedure, the microcatheter used to inject Onyx was cut at the level of jugular sheath.

Preoperative baseline functional status was determined using the modified Rankin scale score (mRS). The same evaluation was made on post-operative days 2, 7, and 30, and at 3, 6, and 12 months. Occlusion of the AVM nidus was categorized as complete (no residual nidus) and near complete obliteration (residual nidus < 3 mm in diameter).

Procedure safety was evaluated by assessing the periprocedural complications occurring within 1 month after embolization (12). Any deficit that resolved within first 30 days was characterized as transient. Any deficit that persisted beyond 30 days was considered permanent. An mRS score of ≤ 2 indicated a non-disabling deficit. An mRS score of ≥ 3 indicated a disabling deficit. Periprocedural-related death was defined as any death occurring within 30 days after the procedure.

### Statistical Analysis

SPSS 22.0 was used for statistical analysis. Measurement data were expressed as mean ± standard deviation if complied with normal distribution or median and quartile if non-normal. Fisher's exact probability method was used to analyze the effects of the deep draining vein, angle of venous draining into the sinus, location in the eloquent area, and size of nidus on perioperative complications of AVMs treated by TVE. *P* < 0.05 was considered statistically significant (two-sided).

## Results

### Baseline Data

Clinical and imaging data of 27 patients with ruptured AVMs treated by TVE from November 2016 to May 2020 were included in this prospective study. The cohort comprised of 19 (70.4%) males, and 8 (29.6%) were females, aged 7–59 (mean age: 29 ± 15) years. The Spetzler–Martin classification before TVE was as follows: I for 4 cases, II for 5 cases, III for 15 cases, and grade IV for 3 cases. A total of 27 lesions were located on the supratentorial region; these included 10 cases with aneurysm, 1 with venous bulb, and 5 with stenosis of draining vein. The mean size of the nidus preoperatively was 2.85 ± 1.36 cm.

### Surgical Results

Among the 27 patients with AVMs, 25 (92.6%) were successfully treated by TVE, and 2 who failed were treated by transarterial embolization. Subsequently, 15 (60%) patients with AVMs underwent TVE alone, 8 (32%) patients received transarterial embolization before TVE, 1 (4%) patient received craniotomy, and 1 (4%) patient underwent SR after TVE, 2 (8%) patients underwent craniotomy and stereoradiotherapy. Of the 25 patients successfully treated with TVE, 22 (88%) were completely cured as confirmed by imaging, and three patients had partial residual nidus.

### Complications

A total of seven cases with complications were noted in this group (Table 1), including three cases with intraoperative hemorrhage (two cases related to increased nidus pressure during pressure cooker, one case related to venous rupture), three with postoperative hemorrhage (two cases due to residual nidus, one case related to increased nidus pressure during pressure cooker), and one with postoperative cerebral infarction.

**Table 1:** Characteristics of patients with complications and follow-up results.

		**Spetzler-Martin** **grade**			**Nidus size** (**cm**)						**mRS score**		
**Case**	**Sex/age (yrs)**	**Initial**	Pre^*^	**Location/side**	**Initial**	Pre^*^	**Venous drainage**	**Arterial feeders**	**Type of complication**	**Time of complication**	Pre^*^	**At discharge**	**Score/Month**	**Imaging** **follow-up**
1	M/59	III	III	BG/R	2.8	2.8	VG, CCV	PCA, PcoA	IVH	Intraoperative	1	1	1/34	Cure
2	F/31	III	III	BG/L	1.9	1.9	VG	ACA, AcrA, PCA	IVH	Intraoperative	3	2	0/24	Cure
3	M/31	III	III	T/R	5.5	4.3	CCV	AChA	Vein rupture	Intraoperative	2	3	0/6	Cure
4	F/29	V	IV	Corpus Callosum	3.5	2.1	VG	ACA, PCA, AcoA	IVH	At 1 day postoperatively	1	1	0/34	Cure
5	F/49	II	II	Thala mus/R	1.2	1.2	CCV	PCA	THbiV	At 11 h postoperatively	4	5	0/34	Cure
6	M/28	III	III	BG/R	1.4	1.4	VG	AcrA	IVH	At 2 day postoperatively	1	5	NA/22	NA
7	M/8	III	III	Dience phalon	2.5	2.5	VG	PCA	Encephaled-ema, Thalamic infarction	At 1 day postoperatively	1	5	5/16	NA

* *Before transvenous endovascular embolization*.

ACA, anterior cerebral artery; AChA, anterior choroidal artery; AcoA, arteriae communicans anterior; AcrA, anterior choroidal artery; BG, basal ganglia; CCV, cortical cerebral vein; F, female; IVH, intraventricular hemorrhage; L, left; M, male; Pre^*^, before transvenous endovascular embolization; PCA, posterior cerebral artery; PcoA, arteriae communicans posterior; R, right; T, temporal; THbiV, thalamic hemorrhage breaks into ventricles; VG, vein of Galen; NA, not available.

**Figure 1:**
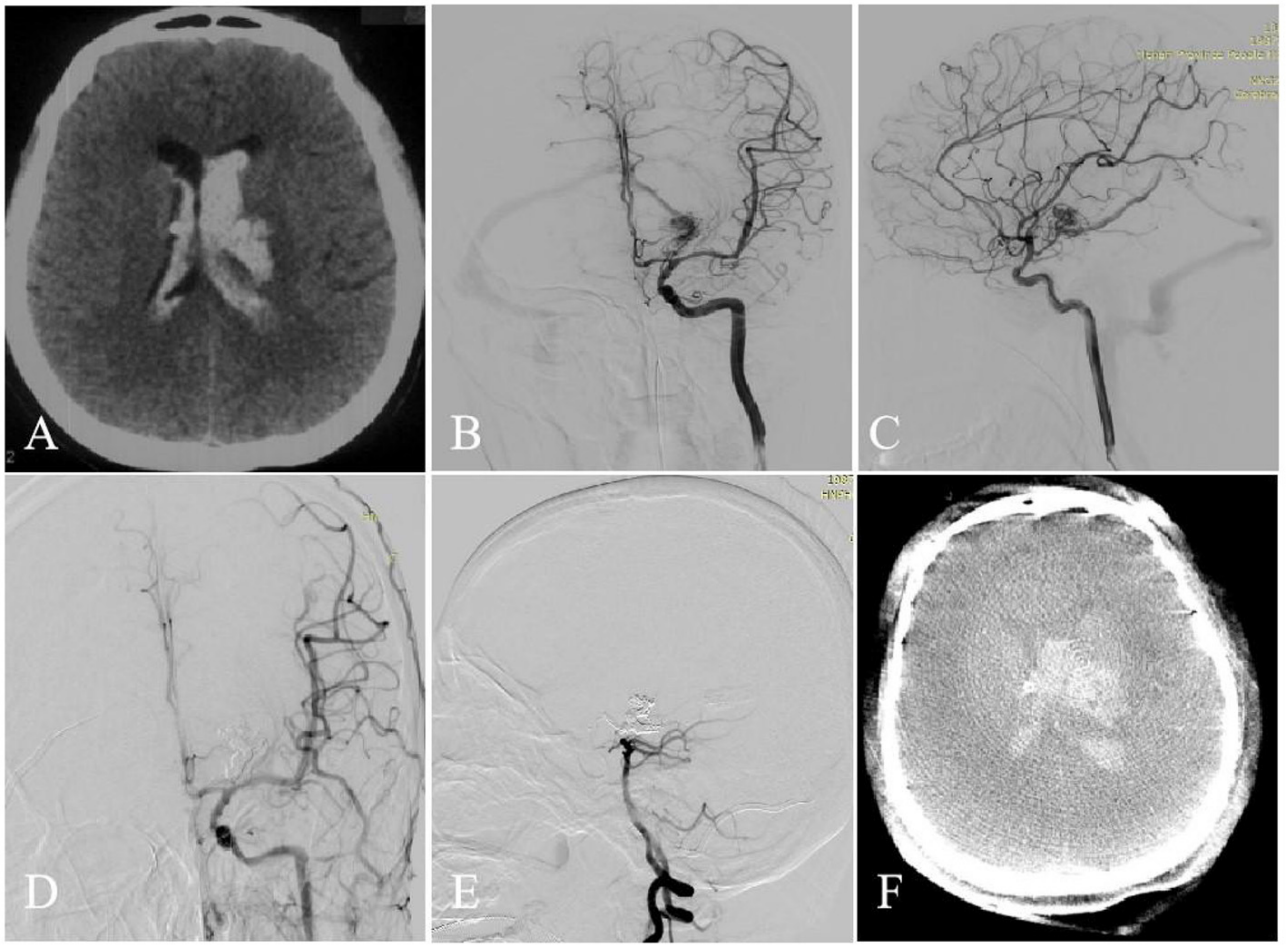
A 31-year-old woman with left basal ganglia AVM. Intraventricular hemorrhage occurred (**A**, CT). Selective DSA of the left ICA [anteroposterior **(B)** and lateral **(C)** views] demonstrated that the AVM is fed by the ACA, AcrA, and PCA and drained a single venous outlet via the VG. At the end of the operation, the AVM did't appear at the last angiography [anteroposterior **(D)** and lateral **(E)** views]. Intraoperative intraventricular hemorrhage occurred (**F**, Dyna CT).

**Figure 2:**
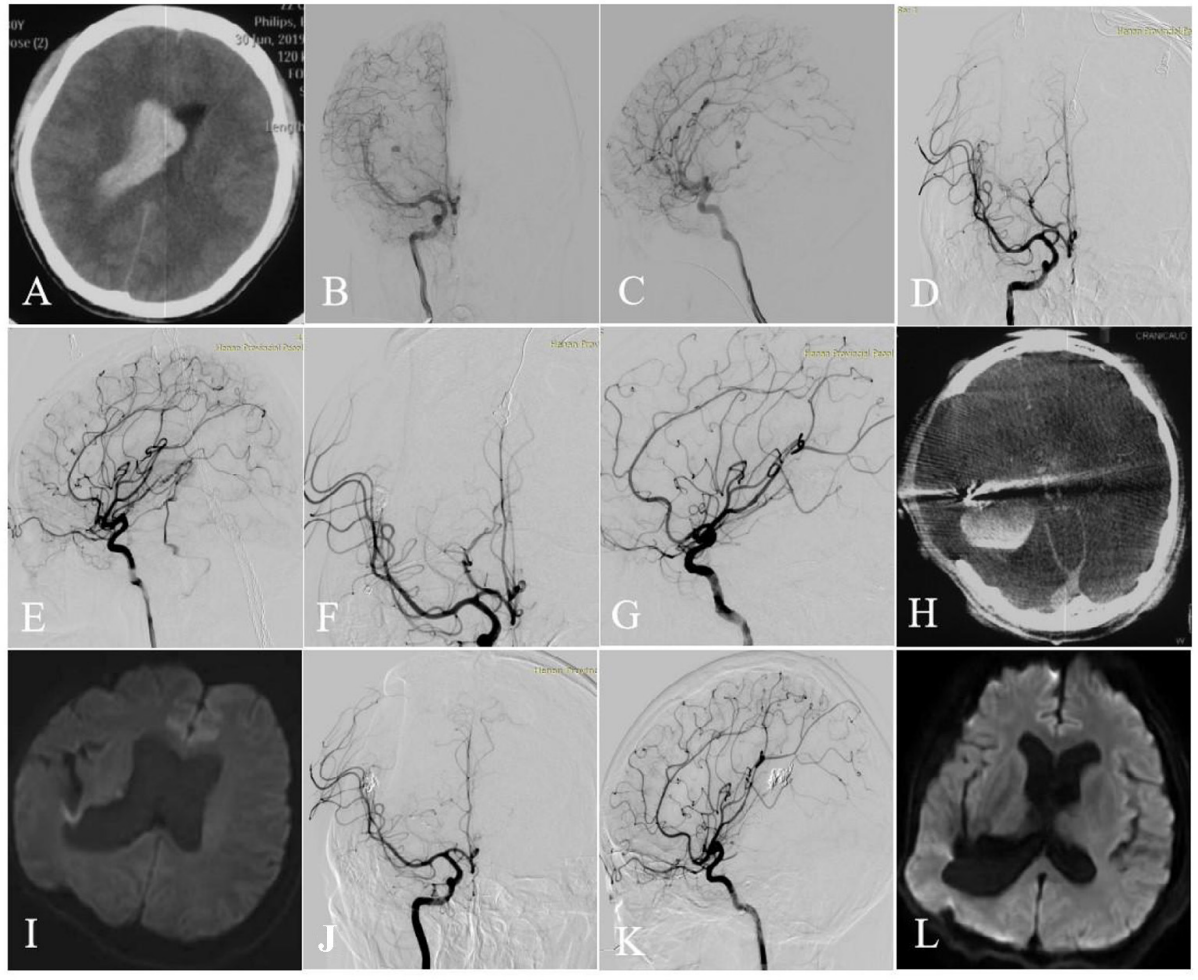
Axial CT image showed intraventricular hemorrhage **(A)**. Selective DSA of the right ICA [anteroposterior **(B)** and lateral **(C)** views] showed that the AVM with an aneurysm (flow related pedicle) located at the temporal lobe was fed by the branches and perforators of ACA, MCA, and ICA and drained a single venous outlet via the cortical cerebral vein. After 3 months, TVE was performed. First, targeted embolization of the aneurysm [anteroposterior **(D)** and lateral **(E)** views]. And then the AVM was completely angiographically obliterated at the end of the operation [anteroposterior **(F)** and lateral **(G)** views]. Intraoperative intracerebral hemorrhage occurred (**H**, Dyna CT). No cerebral infarction was seen on Axial DWI performed 3 days **(I)** after surgery and then 6 months **(L)**. The AVM was completely angiographically obliterated at the 6-month follow-up [anteroposterior **(J)** and lateral **(K)** views].

One case had intraventricular hemorrhage (due to residual nidus) confirmed by cranial CT on the second day postoperatively (case 6) in a shallow coma state, with left limb paralysis. Although trepanation and drainage, lumbar cistern drainage, and symptomatic treatment were performed, the patient's symptoms did not improve. The other case, which had been described in the previous studie (6), was in a shallow coma state on day 1 postoperatively (case 7), and cranial MRI showed cerebral edema and acute cerebral infarction in the left thalamus, which might be caused by the use of anhydrous ethanol embolization agent. The patient was administered conservative drug treatment and presented mRS score of 5 at discharge. One patient showed thalamic hemorrhage breaking into the ventricle due to malformation rupture preoperatively (case 5). On day 1 postoperatively, the patient suddenly presented nausea, vomiting, and a declining consciousness level. Cranial CT showed that cerebral hemorrhage was increased than before. Trepanation and drainage was performed in the Emergency Department. Postoperatively, the legal representative asked the patient to be transferred back to the local hospital for conservative treatment. Another patient (case 4) showed hemorrhage in the fourth ventricle and posterior horn of bilateral lateral ventricle on day 1 postoperatively, which was caused by residual nidus, but the symptoms were not evident. The symptoms improved after symptomatic treatment with dehydration, reducing intracranial pressure, and other drugs. The intraoperative intraventricular hemorrhage occurred in two patients, and conservative medical treatment was performed in 1 patient (case 1) with an mRS score of 1 at discharge. Another case (case 2) underwent bilateral trepanation, and drainage, and symptomatic treatment with drugs. The symptoms improved at discharge than at admission, with the mRS score of 2 (Figure 1). Case 3 underwent a craniotomy to remove a part of hematoma and resect the part of malformation lesions before the transvenous approach. After 3 months, the TVE was performed. Intraoperative venous rupture related to increased venous pressure during pressure cooker and hemorrhage occurred (Figure 2). Symptomatic drug treatment was given postoperatively with an mRS score of 0 at 30 days postoperatively.

Of the seven patients with perioperative complications, two cases exhibited disabling or fatal complications. No long-term complications occurred in any of the patients during follow-up.

### Influencing Factors of Complications

Univariate analysis showed no statistical significance in sex (*P* = 0.355), age (*P* = 0.656), size of nidus (whether or not ≥ 3 cm, *P* = 0.659), eloquent area (*P* = 0.137), location of nidus in the deep part (*P* = 0.637), type of draining vein (*P* = 0.202), the number of venous drainage (*P* = 0.49), angle of draining vein entering venous sinus (*P* = 1.0), preoperative mRS score (whether or not mRS ≤ 2, *P* = 1.0), aneurysms (*P* = 0.058), the time between hemorrhage and TVE (*P* = 1.0) or the transvenous pressure cooker technique (*P* = 0.534) (Table 2).

**Table 2:** Complication group vs. no complication group.

**Characteristic**	**Complication group (*n* = 7)**	**No complication group (*n* = 18)**	***P***
Sex			0.355
Male	4 (16.0)	14 (56.0)	
Female	3 (12.0)	4 (16.0)	
Age			0.656
<29	3 (12.0)	11 (44.0)	
≥29	4 (16.0)	7 (28.0)	
Size			0.659
<3 cm	5 (20.0)	10 (40.0)	
≥3 cm	2 (8.0)	8 (32.0)	
Eloquent location			0.137
Yes	7 (28.0)	12 (48.0)	
No	0 (0.0)	6 (24.0)	
Venous drainage			0.202
Deep	5 (20.0)	7 (28.0)	
Superficial	2 (8.0)	11 (44.0)	
The number of venous drainage			0.49
<1.08	6 (24)	17 (68)	
≥1.08	1 (4)	1 (4)	
Location			0.637
Deep	6 (24.0)	13 (52.0)	
Superficial	1 (4.0)	5 (20.0)	
Angle of venous drainage to the sinus			1.0
Acute angle	7 (28.0)	16 (64.0)	
Obtuse angle	0 (0.0)	2 (8.0)	
Preoperative mRS score			1.0
≤ 2	5 (20.0)	11 (44.0)	
>2	2 (8.0)	7 (28.0)	
Aneurysm			0.058
Yes	5 (20.0) 59	4 (16.0)	
No	2 (8.0)	14 (56.0)	
Transvenous pressure cooker technique			0.534
Double catheter	7 (28.0)	15 (60.0)	
Single catheter	0 (0.0)	3 (12.0)	
The time between hemorrhage and TVE			1.0
<63.8 d	4 (16.0)	11 (44.0)	
≥63.8 d	3 (12.0)	7 (28.0)	

## Discussion

The in-depth understanding on the angioarchitecture of AVMs and the continuous development of embolization materials and techniques emphasize that TVE is a promising treatment for AVMs (13). A meta-analysis including 66 AVM patients receiving TVE between 1980 and 2017 reported that the rate of healing embolization was 96.0% (4), which was significantly higher than the traditional transarterial embolization for carefully selected AVMs, such as deep location, small size, ruptured brain AVMs with single draining vein (1, 14). However, while treating AVMs by TVE, surgical complications are a major concern. Perioperative intracranial hemorrhage is considered severe due to its high mortality and morbidity rates. Currently, only a few reports are available on the complications of AVMs treated by transvenous approach.

### Incidence of Complications

A prospective cohort study by Mendes et al. on AVMs treated with transvenous approach reported 3/40 (7.5%) patients with perioperative complications (15). A study from the same clinical center as Mendes's study showed that hemorrhagic complications occurred in 8 (14.0%) procedures (16). Another meta-analysis showed that the complication rate of AVMs treated by the transvenous approach was 10% (17). The complication rate in our center was 25.9%, which was higher than that reported in the literature. This phenomenon could be attributed to the following: First, bleeding from ruptured nidus is a high-risk factor for lesion rebleeding. All patients included in this study were those with ruptured nidus preoperatively. In studys by Mendes et al. (15) and De Sousa et al. (16), the bleeding rate of ruptured nidus preoperatively were only 67.5 and 66.7%, respectively. Second, the patients enrolled in the current study had severe symptoms, and 40.7% patients had mRS ≥ 3 preoperatively, which was higher than 25% in the study by Mendes et al. Third, 37% of the patients in this study had nidus size ≥ 3 cm preoperatively, higher than the rates in the studies by Mendes et al. (28.1%) (15), Lv et al. (36.7%) (17), De Sousa et al. (28.1%) (16). Fourth, 77.8% of the nidus were located deep (basal ganglia, thalamus, and cerebellum) in this study, which was higher than the patients with deep AVMs in studys by Mendes et al. (36%) (15) and De Sousa et al. (56.1%) (16). Fifth, the AVMs included in this study were complex, and the percentage of patients with preoperative Spetzler-Martin score ≥ III in this study (66.7%) was higher than that in studys by Mendes et al. (58.5%) (15) and De Sousa et al. (59.6%) (16).

### Risk Factors Affecting the Occurrence of Complications

In this study, the related factors that may cause surgical complications were analyzed. No significant differences were detected in sex, age, size of nidus (whether or not ≥ 3 cm), eloquent area, location of nidus in the deep part, type of draining vein, the number of venous drainage, angle of draining vein entering the venous sinus, preoperative mRS score (whether or not mRS ≤ 2), aneurysms, the time between hemorrhage and TVE or transvenous pressure cooker technique. Due to the small sample size and single-center study, it was difficult to determine whether the above factors have an impact on the occurrence of surgical complications and multicenter and large-sample clinical data are needed for analysis.

### Mechanism of Complications and Preventive Measures

#### Hemorrhagic Complications

In this study, six patients suffered from a perioperative intracerebral hemorrhage, including five cases with intraventricular hemorrhage and 1 case with venous rupture hemorrhage. These could be attributed to the following: ① Microcatheters deep into the nidus penetrating the draining vein lead to venous perforation; ② The hemodynamics of nidus altered markedly after the formation of “pressure cooker.” Blood stasis occurred in dilated draining veins, and increased venous pressure led to venous rupture and bleeding (7); ③ Normal brain tissue around nidus was in a state of hypoxia and hypoperfusion for a prolonged period due to arteriovenous shunt, and the autoregulation was dysfunctional. After TVE, because of the inability to adapt to the dramatic change of perfusion, perfusion pressure breakthrough syndrome occurred, leading to bleeding (18); ④ Because the nidus was large or irregular, the embolic agent could not reverse the complete penetration to the nidus, resulting in residual nidus. In addition, the draining vein was occluded, sharply increasing the blood pressure in the nidus, resulting in rupture and bleeding of the residual lesions.

To prevent cerebral hemorrhage and reduce perioperative complications of transvenous approach, the surgeon should have experience in neurointerventional therapy and an in-depth understanding of the transvenous approach. Also, the surgeon must be familiar with the structure of malformed vessels using 3D DSA reconstruction images. The guidewire and catheter should be operated as gently as possible to reduce the occurrence of bleeding complications intraoperatively. Thus, we concluded that once the draining vein is occluded, complete embolization of the nidus should be carried out to avoid intraoperative cerebral hemorrhage caused by hemodynamic changes attributed to continuous blood supply from the feeding artery to the nidus. Moreover, during embolization, it is necessary to fully block the main arterial feeders and enhance blood pressure control to reduce intraoperative bleeding complications.

Postoperatively, the venous microcatheter can be retracted tentatively and gently, and the microcatheter can be left in the body if necessary. The complications caused by microcatheter indwelling have not been reported, and hence, the safety still lacks effective data to prove. Recently, the use of detachable-tip microcatheters can avoid the risk of microcatheter indwelling, reduce the risk of sticking and evacuating catheters, as well as cerebral hemorrhage. However, the detachable-tip microcatheter has not been used widely, and its safety needs to be further verified by clinical data obtained from large-sample studies. Even though such a microcatheter is applied, it does not have to be forcibly pulled out from the vein.

The transvenous approach should be considered as the ultimate curative embolization for AVMs, such as a large size of the nidus or the complex vascular structure requiring stepwise embolization. The interval between the two operations should be at least 3 months to reduce the risk of bleeding. In addition, the blood pressure of the patients in the intensive care unit needs to be controlled strictly within 48 h postoperatively, conduct a re-examination of cranial CT, and closely observe changes in this condition. After postoperative cerebral hemorrhage occurs, timely symptomatic treatment should be carried out, and trepanation and drainage or craniotomy hematoma removal can be performed if necessary.

#### Ischemic Complications

A few studies have reported the ischemic events after TVE (15). In this study, only 1 case was judged to have an ischemic complication. This patient had a deep lesion with the deep-draining vein, located in a critical functional area; postoperatively, the patient was in a shallow coma and MRI showed multiple cerebral infarctions. Causes of cerebral infarction were considered to be that the patient suffered an unexpected venous infarct due to occlusion of longer venous outflow, which was consistent with that of a previous study (15). And the nidus was mixed with blood vessels maintaining normal brain function and blood supply, which could cause cerebral ischemia symptoms after embolization. Furthermore, the brain tissue at the lesions of case 7 undertook many important functions and played a key role, which required a richer blood supply. Therefore, researchers should be cautious about such types of AVMs.

## Limitations

There are few clinical applications of TVE worldwide at present, the sample size included in this study is small, with a lack of control group; second, this is a single-center study, and the selection bias may affect the results of the study.

## Conclusion

Combining the existing literature and the experience of our group, the cure rate of selective TVE of AVMs was significantly higher than that of traditional therapies (transarterial embolization, craniotomy, SR) in the same population, with an encouraging short-term effect. However, the intraoperative and postoperative complications of TVE of AVMs cannot be ignored. In addition, more effort should be put into predicting and reducing complications of the TVE of AVMs.

## Data Availability Statement

The original contributions presented in the study are included in the article/supplementary material, further inquiries can be directed to the corresponding author/s.

## Ethics Statement

The studies involving human participants were reviewed and approved by the Medical Ethics Committee of Henan Provincial People's Hospital of China [approval number: 2017 (41)]. Written informed consent to participate in this study was provided by the participants' legal guardian/next of kin.

## Author Contributions

YaH preparation, creation and presentation of the published work, specifically critical review, and commentary or revision-including pre or postpublication stages. WB and YiH formulation or evolution of overarching research goals and aims, development or design of methodology, and responsible for ensuring that the descriptions are accurate and agreed by all authors. BX and JX technical support to ensure the safe implementation of surgery. TL oversight and leadership responsibility for the research activity planning and execution and including mentorship external to the core team. XK management activities to annotate and maintain research data for initial use and later reuse. All authors contributed to the article and approved the submitted version.

## Funding

This work was supported by the Scientific and Technological Project of Henan Provincial Health Commission (No. SB201901068).

## Conflict of Interest

The authors declare that the research was conducted in the absence of any commercial or financial relationships that could be construed as a potential conflict of interest.

## Publisher's Note

All claims expressed in this article are solely those of the authors and do not necessarily represent those of their affiliated organizations, or those of the publisher, the editors and the reviewers. Any product that may be evaluated in this article, or claim that may be made by its manufacturer, is not guaranteed or endorsed by the publisher.
